# Developmental validation of an X-Insertion/Deletion polymorphism panel and application in HAN population of China

**DOI:** 10.1038/srep18336

**Published:** 2015-12-14

**Authors:** Suhua Zhang, Kuan Sun, Yingnan Bian, Qi Zhao, Zheng Wang, Chaoneng Ji, Chengtao Li

**Affiliations:** 1 Shanghai Key Laboratory of Forensic Medicine, Institute of Forensic Sciences, Ministry of Justice, P.R. China, Shanghai 200063, P.R. China; 2 State Key Laboratory of Genetic Engineering, Institute of Genetics, School of Life Sciences, Fudan University, Shanghai 200433, P.R. China; 3 Institute of Forensic Medicine, West China School of Basic Science and Forensic Medicine, Sichuan University, Chengdu 610041, P.R.China

## Abstract

InDels are short-length polymorphisms characterized by low mutation rates, high inter-population diversity, short amplicon strategy and simplicity of laboratory analysis. This work describes the developmental validation of an X-InDels panel amplifying 18 bi-allelic markers and Amelogenin in one single PCR system. Developmental validation indicated that this novel panel was reproducible, accurate, sensitive and robust for forensic application. Sensitivity testing of the panel was such that a full profile was obtainable even with 125 pg of human DNA with intra-locus balance above 70%. Specificity testing was demonstrated by the lack of cross-reactivity with a variety of commonly encountered animal species and microorganisms. For the stability testing in cases of PCR inhibition, full profiles have been obtained with hematin (≤1000 μM) and humic acid (≤150 ng/μL). For the forensic investigation of the 18 X-InDels in the HAN population of China, no locus deviated from the Hardy–Weinberg equilibrium and linkage disequilibrium. Since they are independent from each other, the CDP_female_ was 0.999999726 and CDP_male_ was 0.999934223. The forensic parameters suggested that this X-Indel panel is polymorphic and informative, which provides valuable X-linked information for deficient relationship cases where autosomal markers are uninformative.

Short tandem repeats (STRs) and single nucleotide polymorphisms (SNPs) are widely accepted markers for forensic genetics^1^,^2^. However, both of them have some disadvantages for forensic applications, such as comparatively high mutation rate for STRs^1^, or technical difficulties for SNP detection due to the lack of adequate typing equipment in forensic laboratories^2^. Combining the merits of STRs and SNPs, Insertion/Deletion polymorphisms (InDels) have received increased attention during the last few years^3^,^4^. Since InDels derive from a single mutation event occurring with a low frequency, they are genetically quite stable^4^. InDels, as short-length markers, can greatly improve genotyping success with PCR and electrophoresis in most of forensic laboratories.

With the advent of autosomal InDels studies^3^,^4^, this field of research is also focusing on the X chromosome, given its special transmission pattern. X-InDels can provide genetic information in deficient relationship cases and in cases where autosomes are uninformative. For example, when the alleged father is not available, the likelihood of the claimed kinship could be evaluated indirectly with genetic information of X chromosome from his female relatives. And the probability of paternity exclusion can become greater for X-chromosomal markers than for autosomes when female offsprings are involved. Such studies were already performed in some populations: Germany^5^, Somalia and Iraq^6^, Africa, Europe, East Asian and America^7^,^8^, as well as Brazilian Amazon^9^. So far, up to 33 X-InDels were explored^5^,^6^,^7^,^8^,^9^, however, some of them showed poor polymorphic in Chinese HAN population.

China, the world’s most populated country with 1.371 billion in the national population census in 2010, would benefit from research on X-Indels of forensic interest. The HAN population, as the biggest ethnic group, constitutes about 91.51% of the total population and is widespread across the country. Since less data about X-InDels from HAN population have been published, for this study 18 X-InDels (rs3048996, rs5901519, rs363794, rs2308033, rs66676381, rs5903978, rs3080039, rs55877732, rs10699224, rs2308280, rs45449991, rs3215490, rs25581, rs60283667, rs35574346, rs3047852, rs57608175, rs72417152) plus Amelogenin with allelic length variations of 3–12 bp were chosen. Assay design was keyed towards flexibility by only using three fluorescent labels, leaving room for further extension of the assay. Developmental validation studies of the panel and forensic application in the HAN population were explored. While working on deficient relationship cases and cases where autosomes are uninformative, the 18 polymorphic X-InDels can provide helpful genetic information.

## Methods

### Ethics Statement

Human blood samples were collected upon approval of the Ethics Committee at the Institute of Forensic Sciences, Ministry of Justice, China. A written informed consent was obtained form each participant in this study. Animal blood samples were collected with the approval of the Animal Use Committee of the Institute of Forensic Sciences, Ministry of Justice, China. This study was approved by the Ethics Committee of the Institute of Forensic Sciences, Ministry of Justice, China.

### Marker selection

Candidate InDels were chosen with Human Genome Browser of Galaxy system (http://main.g2.bx.psu.edu/) and the National Center for Biotechnology Information (NCBI). The following principles were followed: (i) bi-allelic InDels positioned on the X chromosome, (ii) located in non-coding areas, (iii) allelic length range from 3 to 12 bp and (iv) Minor Allele Frequency (MAF) in East Asia above 0.1. Flanking regions of the selected InDels were obtained from the University of California Santa Cruz Genome Browser (Human February 2009 Assembly; GRCh37/hg19) at http://genome.ucsc.edu/. Sequences were checked for variants and repeat structures which are likely to interfere with primer design or data analysis.

### Primer design and optimization

Primer designing was performed with the Primer Premier v5.0 and Oligo v6.0, by applying the following criteria: PCR product size from 70 to 200 bp; Tm values from 57 to 64 °C and GC content from 30 to 60%. The obtained primer pairs were then checked for non-specific hybridizations in other genome regions with the NCBI Basic Local Alignment Search Tool (BLAST) at http://blast.ncbi.nlm.nih.gov/. The primer pair for each X-InDel locus was initially tested in a singleplex PCR reaction to evaluate the performance with Qiagen Multiplex PCR kit (Qiagen, Hilden, Germany). PCR amplification was carried out on GeneAmp 9700 PCR system (Applied Biosystems, Foster City, CA). Successful PCR products were then Sanger sequenced. After confirming the exact length and sequencing information of candidate InDels, all the markers were then schematically organized by expected amplicon length and assigned into three different dye-labelling fluorochromes (FAM, HEX, TAMRA) (Applied Biosystems, Foster City, CA).

### PCR setup and PCR-based studies

Due to the high quality of InDels to be amplified in the same PCR reaction, the labelled primer-pairs were combined together at 0.1 μM at first. The PCR system was a 12.5 μL reaction volume containing 1× Qiagen multiplex PCR master mix, 1×Q-Solution, 2 μL primer mix and 0.5–2 ng of template gDNA. Thermal cycling conditions consisted of an initial step at 95 °C for 15 min; 30 cycles at 94 °C for 30 s, T_m_ for 90 s, and 72 °C for 90 s; and a final extension at 60 °C for 60 min. For the T_m_ value, 63, 65 and 67 °C were chosen for testing. Cycle 30 was recommended in the manual. PCR products were analyzed by mixing 1 μL of each amplified product with 9 μL in a 17:1 mixture of Hi-Di formamide (Applied Biosystem, Foster City, CA) and SIZ 350 (AGCU Co, China) for capillary electrophoresis (CE). CE was performed on ABI Prism 3130*xl* Genetic Analyzer (Applied Biosystems, Foster City, CA). Genotyping data were analyzed with GeneMapper v3.2.1 software (Applied Biosystems, Foster City, CA). Default setting of peak height (above 200 RFU) was applied for the analysis.

Optimizations of the multiplex assay were performed based on primer concentrations and peak heights in order to achieve an evenly balanced genotyping profile. After the optimizations of the PCR system, 200 HAN individuals (100 females and 100 males) were genotyped with the optimized PCR system. Based on the size information of each allele, Panel and Bin files were programmed.

### Developmental Validation studies

Developmental validation studies were performed following the ‘SWGDAM Guidelines for Validation of Probabilistic Genotyping Systems (final approval on 06/15/2015)’.

Control DNA of 9947A (Applied Biosystems, Foster City, CA) and the prepared set of 20 case samples (5 peripheral blood samples, 5 buccal swabs, 5 hair rooted samples and 5 muscle samples) were tested in two accredited laboratories (Shanghai Key Laboratory of Forensic Medicine, Institute of Forensic Sciences, Ministry of Justice, China; State Key Laboratory of Genetic Engineering, Institute of Genetics, School of Life Sciences, Fudan University, China). In addition all these samples were amplified locus by locus and the PCR products were Sanger sequenced. Sanger sequencing results and genotyping results at the two different laboratories were compared for reproducibility and accuracy.

Artificially degraded DNA samples were prepared by incubating 5 ng control DNA of 9947A in a 10 μL reaction system with 0.5 μL 10×DNase I buffer and 1 U DNase I (Takara, Dalian, China) at 37 °C for 4, 8, 12 and 14 min. These samples were tested in triplicates.

A sensitivity study of the panel is essential to evaluate the ability of generating reliable profiles from a range of DNA quantities. Serial dilutions of control DNA 9947A were analyzed with quantities of 5 ng, 2 ng, 1 ng, 500 pg, 250 pg, 125 pg, 62.5 pg and 31.25 pg in triplicates.

Stability testing for obtaining results from compromised samples was evaluated by amplifying one ng of 9947A containing different concentrations of common forensic inhibitors: haematin and humic acid. The range of concentrations evaluated here were: 100 μM, 500 μM, 1000 μM, 1200 μM, 1500 μM, 1800 μM, 2000 μM and 5000 μM of haematin and 50 ng/μL, 100 ng/μL, 150 ng/μL, 200 ng/μL, 250 ng/μL, 300 ng/μL and 600 ng/μL of humic acid. The testing was performed in triplicates.

DNA from non-human species may be present on forensic evidentiary material; thus, various types of non-human DNA were also tested to evaluate any PCR products of this panel. Five ng of each DNA of prepared from common animal species (chick, duck, cow, dog, sheep, pig, cat, rabbit and rat) and common microorganisms (*Saccharomyces cervisiae, Escherichia coli, Enterococcus faecalis, Fusobacterium nucleatum, Micrococcus luteus, Staphylococcus epidermidis, and Streptococcus salivarius*) were tested in triplicates.

### Investigation of Forensic efficiency

In order to evaluate the forensic efficiency of the final panel for application in the Chinese HAN population, an extensive population study was conducted. 1054 individuals (575 males and 479 females) of HAN participated in the study. Human genomic DNA (gDNA) was extracted with QIAamp DNA Blood Mini Kit (QIAGEN, Hilden, Germany). The quantity of gDNA was determined by the Quantifiler Human DNA Quantification Kit on the 7500 Real-time PCR System (Applied Biosystems, Foster City, CA). DNA was amplified with the validated panel and electrophorezed on the ABI Prism 3130*xl* Genetic Analyzer (Applied Biosystems, Foster City, CA). Genotyping data were analyzed with GeneMapper v3.2.1 software (Applied Biosystems, Foster City, CA).

Since two alleles were observed for females and only one allele was observed for males at each locus, the testing of Hardy–Weinberg Equilibrium (HWE) and Linkage Disequilibrium (LD) were performed with SNPAnalyzer 2.0^10^ based on the data obtained from the female HAN population (N = 479). Estimation of allelic frequencies was performed by the single counting method. Pearson's chi-squared test for differentiation between females and males was performed with SPSS. Forensic parameters including polymorphism information content (PIC)^11^, power of discrimination in females (PD_F_)^12^, power of discrimination in males (PD_M_)^12^, mean exclusion chance in father-daughter duos lacking maternal genotype information (MEC_D_)^12^, mean exclusion chance in trios involving daughters (MEC_T_)^13^ were computed based on allelic frequencies.

### Quality control

The main experiments were conducted at the Forensic Genetics Laboratory of Institute of Forensic Science, Ministry of Justice, P.R. China, which is an accredited laboratory (ISO 17025), in accordance with quality control measures. All the methods were carried out in accordance with the approved guidelines of the Institute of Forensic Sciences, Ministry of Justice, P.R. China.

## Results and Discussion

18 X-InDels with allelic length variations of 3–12 bp were finally selected from an initial candidate list of 61 markers for incorporation into the panel (detailed information in Table 1). Information regarding the optimized primers, the dye labels, the PCR amplicon lengths and the final concentrations in primer mixes are listed as Supplementary Table S1. Primers for each locus were initially tested in a singleplex PCR reaction to evaluate the performance. The criteria for primer “failure” are defined as those that produce profiles that exhibit incomplete adenylation, the presence of PCR artifacts, low signal, nonspecific products, or no PCR products at all^14^. Once the successful primers at each locus were determined, those were equally combined together for a primer mix of 0.1 μM at first. Based on the results of genotyping profiles, the optimization of each primer’s concentrations in the final primer mix was performed. Then, the primer mixes were tested from 0.1 to 0.8 μM, and final concentrations of each optimized primer are listed in Supplementary Table S1.

The final optimized PCR conditions were an initial denaturation step of 95 °C for 15 min; 30 cycles at 94 °C for 30 s, 65 °C for 90 s, and 72 °C for 90 s; and a final extension at 60 °C for 60 min with the MAX mode of GeneAmp 9700 (Applied Biosystems, Foster City, CA). Three T_m_ values were tested in triplicates with 0.5 ng of control DNA 9947A and the optimal annealing temperature was 65 °C (Supplementary Fig. S1). The average percentages of detected alleles were 93.03% ± 3.060 at 63 °C and 98.27% ± 3.002 at 67 °C, respectively.

200 samples of Shanghai HAN individuals (100 females and 100 males) were amplified with the 18 X-InDel optimized PCR system and CE by 3130*xl* Genetic Analyzer (Applied Biosystems, Foster City, CA). The detailed size information of each allele was collected. Meanwhile these samples were amplified locus by locus with the primers listed in Supplementary Table S1, and the PCR products were then Sanger sequenced. After confirming the accurate sequence of each allele in each sample, the average fragment size and standard deviation for each allele were calculated (Supplementary Fig. S2). The largest 3x standard deviation was 0.3893. Based on these data, the Panel and Bin files were programmed. All the genotyping files of the 200 samples were analyzed with the programmed Panel and the Bin file. 4883 sample alleles from the 200 samples were within ±0.5 bases of a corresponding allele in the Bin file. The results above show that the Panel and Bin file can reliably determine the genotypes and accurately detect the microvariant alleles. In other words, sample genotypes analysed with the Panel and Bin file were rarely sized outside of the Bin range.

Consistent studies, performed at two different accredited laboratories with the prepared samples (control DNA of 9947A and a set of 20 case-samples), demonstrated the reproducibility of the X-InDels Panel (date not shown). And the Sanger sequencing information at each locus of these samples was matched with the obtained genotyping results.

**Figure 1:**
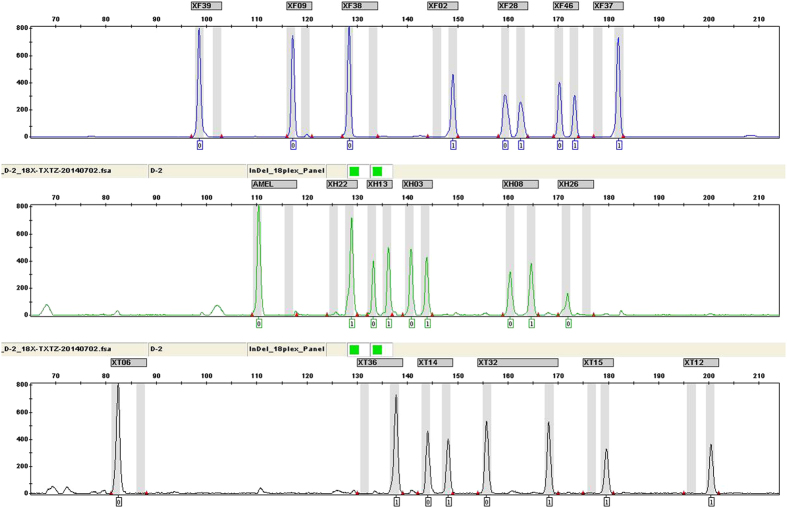
Electropherogram of one human muscle sample DNA (0.5 ng) amplified by the novel 18 X-InDel and Amelogenin panel.

For the set of 20 prepared samples, 4 kinds of routine forensic samples (peripheral blood, buccal swab, hair root and muscle samples) were all genotyped with ideal profiles, indicating that the panel is suitable for typically encountered biological materials in forensic laboratories. The electropherogram of one human muscle sample DNA (0.5 ng) which was amplified with the 18 X-InDels panel is shown in Fig. 1.

**Figure 2:**
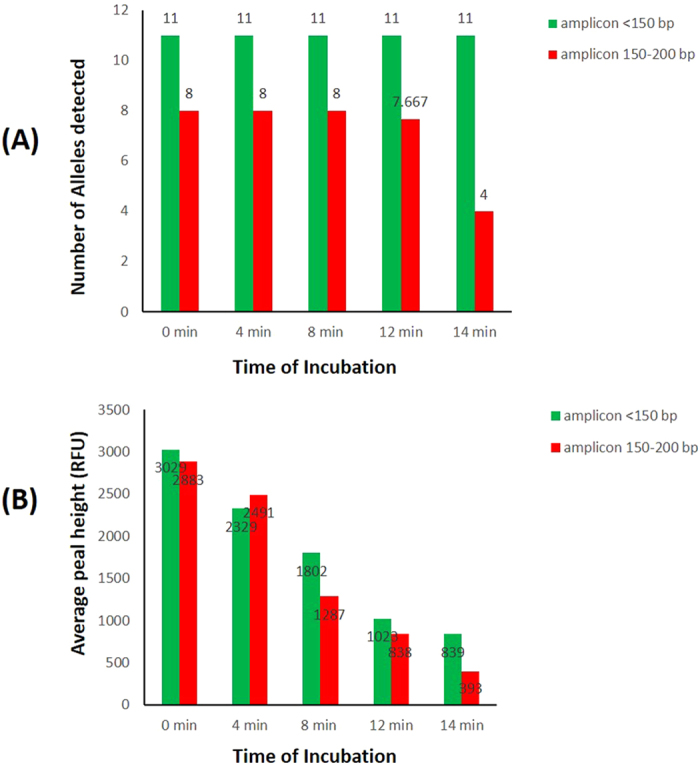
Detection information of artificially degraded DNA by digesting at different time points. (**A**) The average number of alleles detected. (**B**) The average detected peak height.

Apart from the routine samples mentioned above, DNA may be damaged and destroyed in adverse environmental conditions. Environmental exposure degrades DNA molecules by randomly breaking them into smaller pieces. For this InDel panel, PCR amplicons were all within 200 bp. To determine the efficiency of amplification with degraded samples, artificially degraded DNA was tested by digestion at different time points. One ng of undigested 9947A was used as positive control. The results (Fig. 2) show that with increasing time of incubation, the reduction of allele detection rate and peak height, which began with relatively larger amplicons, was observed. This was consistent with the law of degraded DNA. For samples incubated for 8 min, complete profiles were obtained with reduced peak heights for all loci compared to the positive control. For the 12 min incubation, only one allele dropout was observed at XT12 (rs60283667) in one profile. For the 14 min incubation, alleles at 150–200 bp almost lost half while alleles below 150 bp were still all detected with average peak height of 839 RFU.

**Figure 3:**
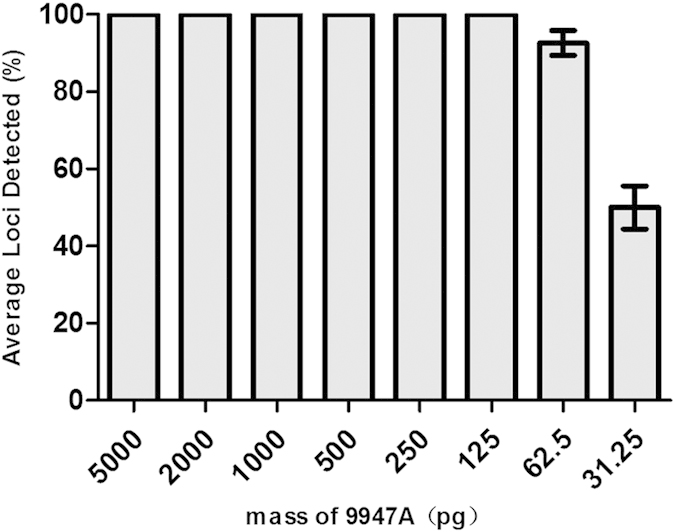
Sensitivity test of template DNA ranging from 5 ng to 31.25 pg. Average percent of loci detected was against DNA template mass (pg). Error bars represent the plus and minus standard deviations.

Sensitivity testing was helpful for determining the upper and lower limits of the panel and to explain the genotyping results of deteriorated DNA. In this study, serial dilutions of the control DNA 9947A (Applied Biosystems, Foster City, CA) were analyzed with quantities of 5 ng, 2 ng, 1 ng, 500 pg, 250 pg, 125 pg, 62.5 pg and 31.25 pg in triplicates. Complete profiles were obtained with DNA from 5 ng down to 125 pg in 12.5 μL PCR reaction system (Fig. 3). When DNA templates down to 62.5 pg and 31.25 pg, the average loci detection rates were 92.63% ± 3.233 and 50.03% ± 5.550, respectively. Comparatively larger loci (150–200 bp) were typically the first to drop out. The results in Supplementary Fig. S3 show the intra-locus balance ratio for each testing. The parameter of Intra-locus balance measures the balance of heterozygous alleles, calculated as the lower peak height divided by the higher peak height for each locus. Intra-locus balance greater than 70% is desired to ensure accurate heterozygote genotyping for a range of template amounts and to facilitate mixture interpretation^15^. When the DNA ranges from 5 ng to 62.5 pg, the intra-locus balance of tested samples was all above 70%. For DNA templates ranging from 2 ng to 500 pg, the intra-locus balance was for all above 80%. However, when using 5 ng DNA for detection, strong signals or pull-up peaks resulting from ineffective fluorescence correction were observed. These results indicated the optimal DNA mass for PCR amplification.

To obtain data for DNA recovered from common inhibitors (hematin and humic acid), a stability test was also performed. The results demonstrated that this panel could tolerate hematin and humic acid in certain concentration ranges. Supplementary Fig. S4-1 and Supplementary Fig. S4-2 illustrate that average percentage of detected loci was decreased when the concentrations of inhibitor increased. As seen in Supplementary Fig. S4-1, full profiles were obtained with hematin ≤1000 μM. When hematin increased to 2000 μM, the average loci detection rate was 33.30% ± 8.052. When the hematin increased to 5000 μM, the DNA profile presented a negative one. In Supplementary Fig. S4-2, full profiles were obtained with humic acid ≤150 ng/μL. When humic acid increased to 200 ng/μL, the average loci detection rate was 64.97% ± 3.060 and when humic acid increased to 300 ng/μL, the average loci detection rate was 36.80% ± 5.300. Finally, for humic acid increased to a concentration of 600 ng/μL, the DNA profile presented a negative one.

For the specificity test, all the genotyping results from common animal species and the microbial pool (closely related with human activity) showed that no reproducible peaks above 50 RFU except for rat. A peak at 137.64 bases with 671 RFU, occurred between XF38 and XF02 and was detected in the profile of rat DNA genotyped with this panel (Supplementary Fig. S5).

For the forensic investigation of the 18 X-InDels, no significant deviation from HWE expectations was detected in the distribution after Bonferroni correction among the HAN female population (N = 479). Data from female and male samples were not significantly different in any of the examined loci by Pearson's chi-squared test with SPSS (P = 0.777). Since no significant difference was found between the genders, the allelic frequencies were calculated by pooling female/male data into one group. For the HAN population, 1054 individuals were investigated. Allelic frequencies and forensic parameters are listed in Table 2. Based on the data from female HAN individuals (N = 479), the LD analysis of 18 X-InDel was explored. The parameters for LD analysis were: MAF threshold is 0.1, min haplotype frequency is 0.01, MAX segment limit is 500 K and r^2^ threshold is 0.8. By pairwise LD calculation and Gabriel’s method^10^, the results (Supplementary Fig. S6) showed no LD was existed among the 18 X-InDels. Since all of the 18 InDels were independent from each other, the combined forensic efficiency parameters were calculated based on allelic frequencies while Cumulative Discrimination Power for females (CDP_female_) was 0.999999726 and Cumulative Discrimination Power for males (CDP_male_) was 0.999934223 in the HAN population. These results show that the 18 polymorphic and informative X-InDels included in this panel can provide helpful genetic information in HAN population.

## Conclusions

In this paper, the developmental validation of a novel X-InDel panel and the application information in HAN population of China are reported. 18 X-InDels plus Amelogenin with allele length variations of 3–12 bp were co-amplified in a single PCR reaction system. The studies of developmental validation demonstrated that the panel is a robust, accurate, specific and sensitive tool. The statistical parameters of forensic importance for this 18 X-InDel system in HAN population showed adequate polymorphism and independence between the tested loci for human identification purposes. The power of discrimination estimated for this genetic system in the HAN population provides useful X-linked information to establish deficient relationship cases. Also, the new data obtained in this study would be useful for enriching the X-InDel databases.

## Additional Information

**How to cite this article**: Zhang, S. *et al.* Developmental validation of an X-Insertion/Deletion polymorphism panel and application in HAN population of China. *Sci. Rep.* **5**, 18336; doi: 10.1038/srep18336 (2015).

## Supplementary Material

Supplementary Information

## Figures and Tables

**Table 1:** General information of 18 X-InDels included in the studied panel.

InDel Marker	Locus Name (in the panel)	Physical Location^a^ (bp)	Genetic Distance^b^ (cM)	Length Detail	Genotyping Result^c^ (9947A)
rs3048996	XF02	10234839	18.8227	-/ATC	I/D
rs55877732	XH03	12572196	23.3022	-/AGA/GAA	I/D
rs25581	XT06	12912862	24.2560	-/TGAGA	I/D
rs10699224	XH08	13711300	26.9184	-/GTTA	D
rs5901519	XF09	13809001	27.2506	-/ACC	I
rs60283667	XT12	28984077	43.3920	-/TCAC	I
rs2308280	XH13	29157973	43.7001	-/TTA	I
rs35574346	XT14	37655740	58.2836	-/AAAC	I/D
rs3047852	XT15	38262701	59.0364	-/ATC/ATT	I/D
rs45449991	XH22	76870484	85.5000	-/AAC	I/D
rs3215490	XH26	88009690	88.3116	-/GACA	I
rs363794	XF28	96855747	97.3557	-/ATA	D
rs57608175	XT32	116901988	114.9118	-/GGTCATCACGAG	I/D
rs72417152	XT36	131760173	133.8561	-/GTATAT	I
rs2308033	XF37	135825432	138.5429	-/CTT	I
rs66676381	XF38	137369795	139.8807	-/TTAAA	I/D
rs5903978	XF39	137713260	140.3272	-/GTAA	I/D
rs3080039	XF46	154561961	187.1000	-/TAA	D

^a^Location in the X chromosome according to UCSC Table Browser, table 131.

^b^Distance between the locus and the p terminal of X chromosome.

^c^D: allele of deletion; I: allele of insertion.

**Table 2:** Allelic frequencies and forensic parameters of Chinese HAN population (N = 1054).

InDel marker	locus name	F_I_	F_D_	PIC	HET	PD_F_	PD_M_	MEC_D_	MEC_T_
rs3048996	XF02	0.7913	0.2087	0.2758	0.6606	0.497	0.3303	0.1652	0.4605
rs55877732	XH03	0.5395	0.4605	0.3734	0.9938	0.6234	0.4969	0.2484	0.5613
rs25581	XT06	0.1500	0.8500	0.2225	0.5100	0.4125	0.2550	0.1275	0.3883
rs10699224	XH08	0.6582	0.3418	0.3487	0.8999	0.5962	0.4499	0.7200	0.5405
rs5901519	XF09	0.4762	0.5238	0.3744	0.9977	0.6244	0.4989	0.2494	0.5621
rs60283667	XT12	0.6477	0.3523	0.6520	0.9127	0.8634	0.6965	0.5128	0.7297
rs2308280	XH13	0.4958	0.5042	0.3750	0.9999	0.6250	0.5000	0.2500	0.5625
rs35574346	XT14	0.7919	0.2081	0.2753	0.6592	0.4962	0.3296	0.1648	0.4598
rs3047852	XT15	0.6301	0.3699	0.3575	0.9323	0.6063	0.4661	0.2331	0.5483
rs45449991	XH22	0.3412	0.6588	0.3485	0.8991	0.5960	0.4495	0.2248	0.5403
rs3215490	XH26	0.6125	0.3875	0.3620	0.9494	0.6114	0.4747	0.2373	0.5522
rs363794	XF28	0.5532	0.4468	0.3722	0.9887	0.6221	0.4943	0.2472	0.5603
rs57608175	XT32	0.3268	0.6732	0.3432	0.8800	0.5896	0.44	0.22	0.5354
rs72417152	XT36	0.8728	0.1272	0.1974	0.4441	0.3701	0.222	0.111	0.351
rs2308033	XF37	0.8584	0.1416	0.2135	0.4862	0.3975	0.243	0.1215	0.3751
rs66676381	XF38	0.1089	0.8911	0.1753	0.3882	0.3317	0.1941	0.0971	0.3166
rs5903978	XF39	0.1592	0.8408	0.2318	0.5354	0.4279	0.2677	0.1338	0.4016
rs3080039	XF46	0.2603	0.7397	0.3109	0.7702	0.5477	0.3851	0.1925	0.5021

F_I_: Frequence of Insertion Allele; F_D_: Frequence of Deletion Allele; PIC: polymorphism information content;

HET: Heterozygosity; PD_F_: power of discrimination in females; PD_M_: power of discrimination in males;

MEC_D_: mean exclusion chance in father-daughter duos; MEC_T_: mean exclusion chance in trios involving daughters.
