# Supplementary material for: Developmental validation of an X-Insertion/Deletion polymorphism panel and application in HAN population of China
Source: Sci Rep. 2015 Dec 14;5:18336. doi: 10.1038/srep18336 (PMC4677316; doi:10.1038/srep18336)
Supplement: Supplementary Information [file srep18336-s1.pdf]

Supplementary Figures S1-S6 and Supplementary Table S1

Developmental validation of an X-Insertion/Deletion polymorphism panel and application in HAN population of China

Suhua Zhang[1,2 +], Kuan Sun[3+], Yingnan Bian[1], Qi Zhao[1], Zheng Wang[1], Chaoneng Ji[2], Chengtao Li[1]*

[1] Shanghai Key Laboratory of Forensic Medicine, Institute of Forensic Sciences, Ministry of Justice, P.R. China, Shanghai 200063, P.R. China

[2] State Key Laboratory of Genetic Engineering, Institute of Genetics, School of Life Sciences, Fudan University, Shanghai 200433, P.R. China

[3] Institute of Forensic Medicine, West China School of Basic Science and Forensic Medicine, Sichuan University, Chengdu 610041, P.R.China

***** Corresponding author: lichengtaohla@163.com

[+] These authors contributed equally to this work

Shanghai Key Laboratory of Forensic Medicine, Institute of Forensic Sciences,

Ministry of Justice, P.R. China, Shanghai 200063, P.R. China

E-mail: lichengtaohla@163.com; Phone: +86-21-52351327; Fax: +86-21-52352959.

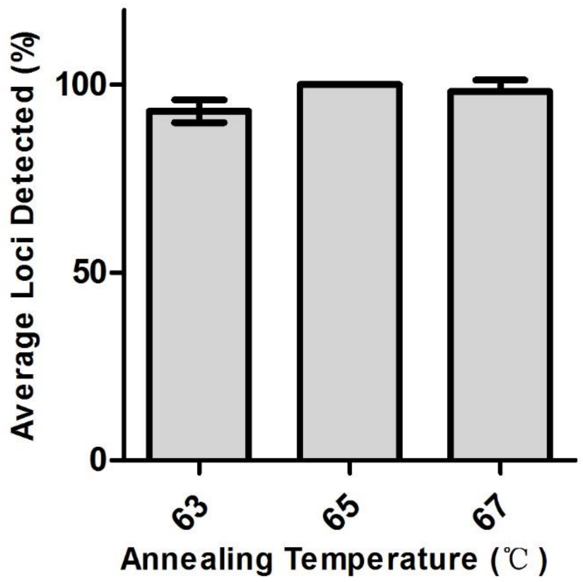

Supplementary Fig. S1. Annealing temperature at 63℃, 65℃ and 67℃ of the novel 18 X-InDel Panel by amplifying 0.5 ng of control DNA of 9947A. Average percent of loci detected was against annealing temperature. Error bars represented the plus and minus standard deviations in triplicate.

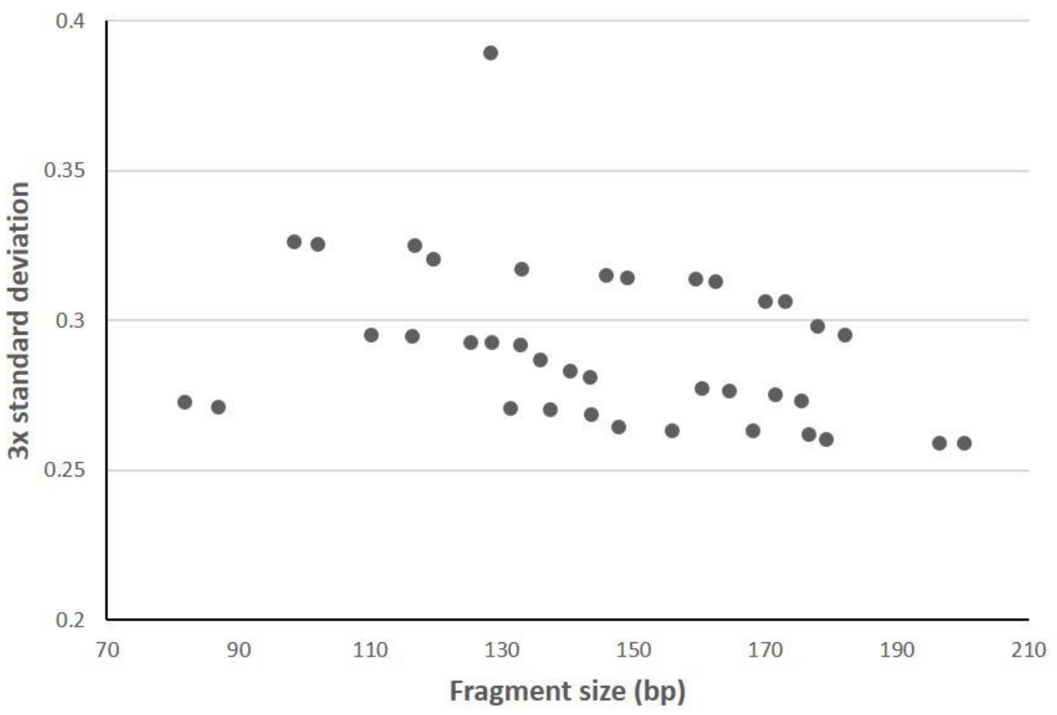

Supplementary Fig.S2. The average fragment size against 3x standard deviation for each allele.

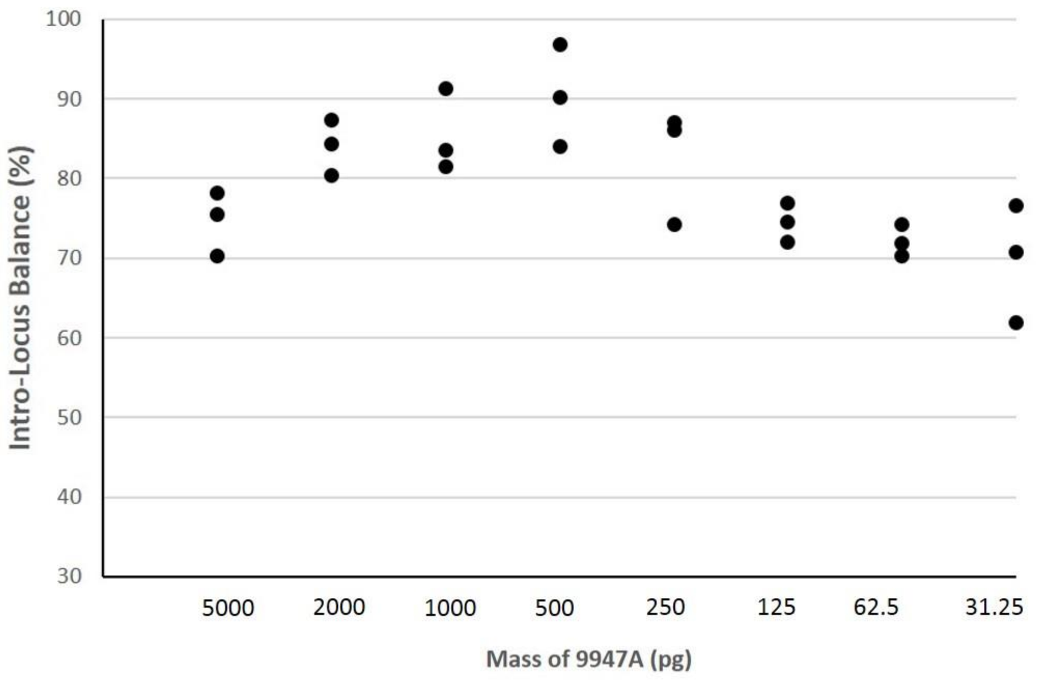

Supplementary Fig. S3. Intro-locus balance information of template DNA ranging from 5 ng to 31.25 pg.

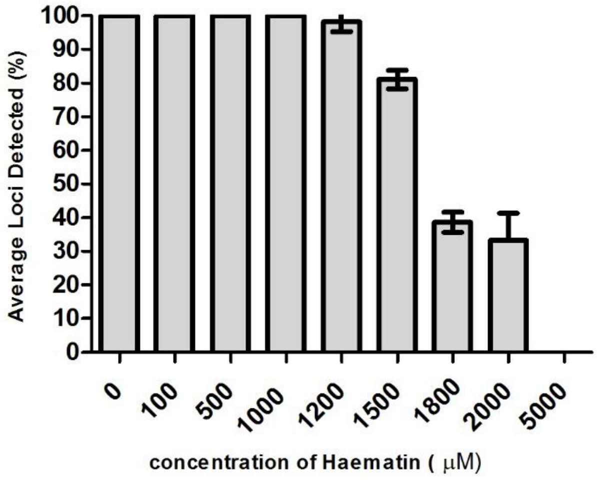

Supplementary Fig.S4-1 Stability testing of haematin by amplifying 1 ng of control DNA of 9947A in the presence of increasing amounts of haematin.

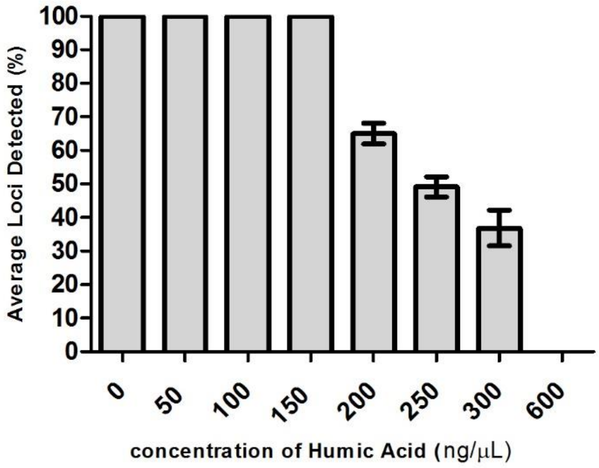

Supplementary Fig.S4-2. Stability testing of humic acid by amplifying 1 ng of control DNA of 9947A in the presence of increasing amounts of humic acid.

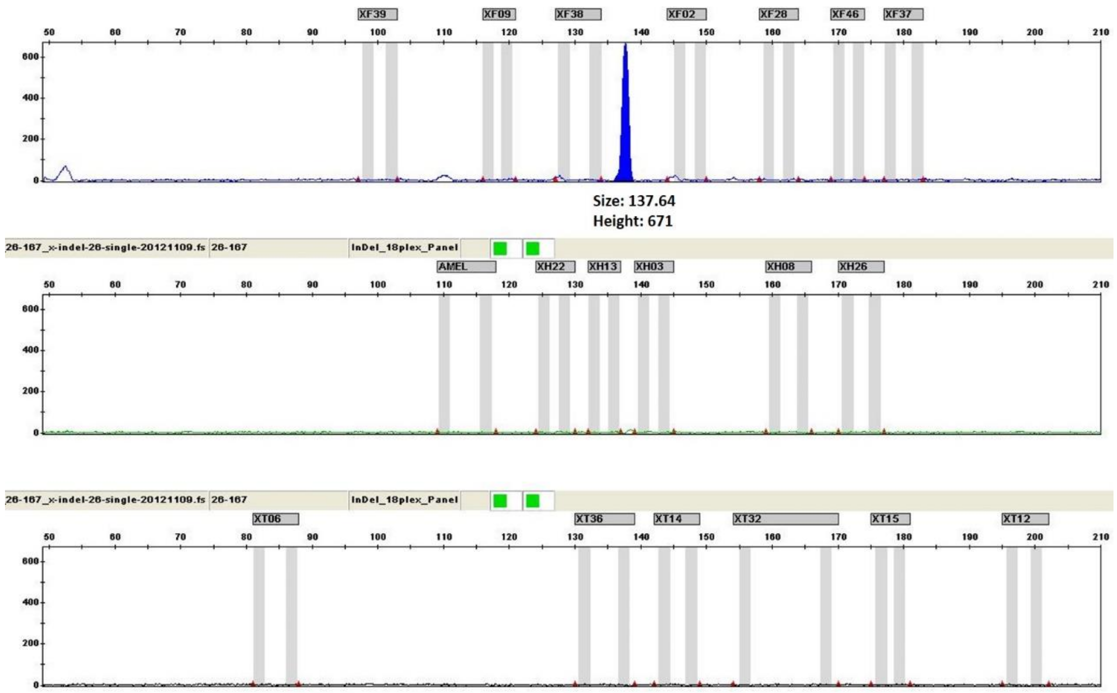

Supplementary Fig.S5. Genotyping result of 5 ng rat DNA by 3130xl Genetic Analyzer. A peak (size: 137.64, Height: 671 RFU) was detected.

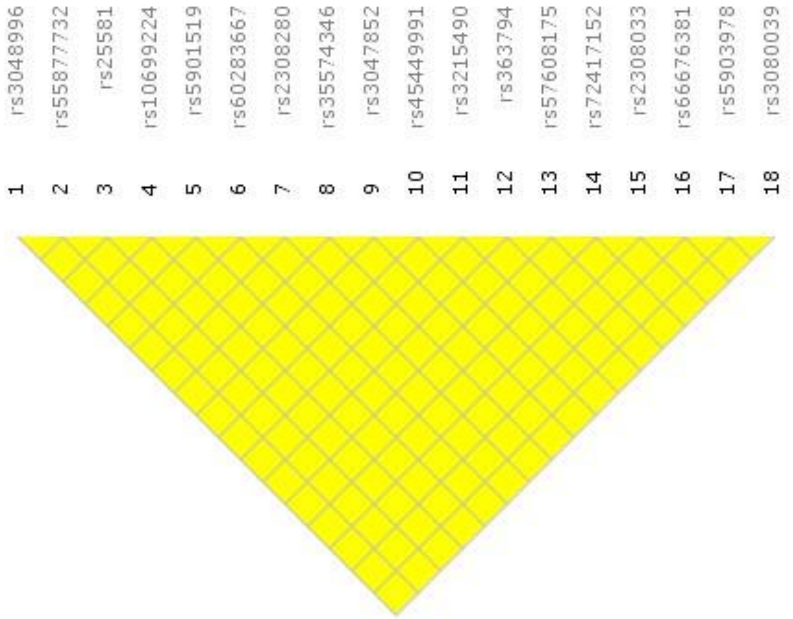

Supplementary Fig. S6 LD analysis of 18 X-InDels among Female HAN population (N=479) by SNPAnalyzer 2.0 software. (Yellow color in the linkage disequilibrium pattern graph means no pairwise LD existed between adjacent SNPs; Red color means pairwise LD existed between adjacent SNPs).

Supplementary Table S1. Detail primer Information of the 18 X-InDels of the studied panel

InDel Marker	Locus Name	Dye lable	Primer sequence:(5'-3')	Primer concentration (μM)	Tm (℃)	Fragment size range (bp)
rs3048996	XF02	FAM	F: TGAATATACTGCAGGGTTCTGTTACAAC	0.20	62	146-149
			R: AGATAGACAGGAGATGAGTGAATGGCT	0.20	63	
rs55877732	XH03	HEX	F: ACCCTAGTTCTACAAAGCGCAAGTC	0.20	63	141-144
			R: AAACATTTTTCAAGGGCAATGATGT	0.20	56	
rs25581	XT06	TAMRA	F: CCTTTCTTGCACATTCTAGCTGAAC	0.20	61	82-87
			R: GGGGTGGTGACAGAAGGGAAT	0.20	61	
rs10699224	XH08	HEX	F: TGTCACCACATTTCCTTCTGGGTA	0.20	61	159-163
			R: TCACTTCCATTTGGCTTACTTCCTC	0.20	61	
rs5901519	XF09	FAM	F: TTGACGGGAATTGAGTCACCTG	0.16	60	117-120
			R: CTAAGGACAGCCTGAATCCCAGAT	0.16	62	
rs60283667	XT12	TAMRA	F: CCTTATTTTGTGCCTTTTATTCTTGG	0.80	58	197-201
			R: TCCTCTAAATTGGGGACCTATGTGTA	0.80	61	
rs2308280	XH13	HEX	F: CTACCAACAAAATCCATTCTGGAATAA	0.60	59	133-136
			R: GTTTTATAGCAACACAAAATTGACTAAGACA	0.60	60	
rs35574346	XT14	TAMRA	F: ACTGGTAGGATCTGGAACATCTGC	0.20	62	144-148
			R: TGACTGTGGGCTTAAATCAAAACTT	0.20	58	
rs3047852	XT15	TAMRA	F: TTCCCAGATTTGAAATGTATGAAACTCT	1.60	59	177-180
			R: CCTTAGTGCCTTGTTAGAAGGAATGA	1.60	61	
rs45449991	XH22	HEX	F: CCGAGTAGAGCTTAACATTTATACCTG	0.80	62	126-129
			R: AGACATGATTGTGCCACTGGATTT	0.80	59	
rs3215490	XH26	HEX	F: TCATCTATATTGAGTCAGCATTTGAACC	0.40	61	170-174
			R: CAGAATCTCTGGAAACACTTGGTAGAA	0.40	62	
rs363794	XF28	FAM	F: TGGTCTCTGGAGTGCAATTTTAAGTC	0.60	61	160-163
			R: CCAAGCCAGCCATTTGTTCTTC	0.60	60	
rs57608175	XT32	TAMRA	F: CAAATTGACTATAGCCTTCCACCCT	0.20	61	156-168
			R: TGCCTTCCTCTCATTGACTTCATAA	0.20	59	
rs72417152	XT36	TAMRA	F: TGTAAAGCTACACCAATGGACAGATG	0.20	61	131-137
			R: TGCAAAGATTAAGTGCATTTTCTCTG	0.20	58	
rs2308033	XF37	FAM	F: ATGGCATTTAGTCTCAGGTCTGCTTA	0.16	61	179-182
			R: CAACTGGTCCACCCTAACTGTATCC	0.17	64	
rs66676381	XF38	FAM	F: CTTTGGGTGATAGGAGGTTTTGC	0.20	60	128-133
			R: AACCCCTGGTGCTGTGTGTAAAT	0.20	60	
rs5903978	XF39	FAM	F: TGTACCACTGTAAAGCCCCCG	0.20	61	98-102
			R: GCTGCATTGTCTGGCAATGAA	0.20	57	
rs3080039	XF46	FAM	F: ACCTTGGACAAAGTTACTTAGCTGCT	0.16	61	170-173
			R: TAGCAAGTCACACATGGATGAGAAA	0.16	59	
